# Resolubility of eye care in a secondary care center: a quantitative approach

**DOI:** 10.31744/einstein_journal/2019GS4385

**Published:** 2019-02-28

**Authors:** Inês Paula Regina Mainini Oliveira, Silmara Meneguin

**Affiliations:** 1 Universidade Estadual Paulista “Júlio de Mesquita Filho”, Botucatu, SP, Brazil.

**Keywords:** Unified Health System, Diabetes mellitus, Secondary care, Eye health, Sistema Único de Saúde, Diabetes mellitus, Atenção secundária à saúde, Saúde ocular

## Abstract

**Objective:**

To evaluate the resolubility of ophthalmologic care in an integrated health center.

**Methods:**

This was a cross-sectional study including 816 patients who were attended from November 2013 to November of 2015. Data were collected from a medical consultation database and patients’ medical records.

**Results:**

The majority of participants were women, non-diabetic, and had high school education. The main cause of referral for ophthalmologic evaluation was the consultation with a specialist, the waiting time for consultation was shorter for non-diabetic patients.

**Conclusion:**

This Integrated health center presented, partial resolubility conditions to meet the ophthalmologic needs of users of the Brazilian Unified Health System. Eye care needs to be reorganized particularly by consider the priority cases of those at ophthalmological risk, and referrals should be done to the adequate care level and on time to guarantee resolubility.

## RESUMO

**Objetivo:**

Avaliar a resolubilidade do cuidado oftalmológico em um centro integrado de saúde.

**Métodos:**

Trata-se de estudo transversal realizado com 816 pacientes atendidos no período de novembro de 2013 a novembro de 2015. Os dados foram coletados consultando-se o banco de dados institucional e os prontuários.

**Resultados:**

A maioria dos participantes era não diabética, do sexo feminino, e com Nível Médio de ensino. A principal causa de solicitação de avaliação oftalmológica foi a consulta com o especialista, cujo tempo de espera foi menor para os não diabéticos.

**Conclusão:**

Este centro integrado de saúde apresentou, em parte, condições de resolubilidade para atender as necessidades oftalmológicas dos usuários do Sistema Único de Saúde, mas notou-se necessidade de reorganização deste cuidado, considerando prioridades baseadas em critérios de risco oftalmológico, conferindo que o encaminhamento ao nível de atenção deve ser capaz de propiciar, em tempo, a resolubilidade.

## INTRODUCTION

The knowledge about the main problems of public health enables actions with practical and real effects in health services. In this context, epidemiologic profile in Brazil has been changing since the 1950. Currently, non-communicable chronic diseases (NCCD) had assumed a prominent role among main causes of morbimortality,^(^ ^1^ ^,^ ^2^ ^)^ among which metabolic diseases are highlighted in relation to improvements in life conditions of humans and development of new technologies applied to health.^(^ ^3^ ^)^

Of these diseases, the *diabetes mellitus* (DM) deserves emphasis on studies that involve actions and health services needed for population’s integral health assistance because of this disease impact on individuals socioeconomic status and health.^(^ ^4^ ^)^

Complications of DM can be acute. We highlight, among them, hypoglycemia, ketoacidosis and hyperosmolar coma. They can also be chronic such as nephropathy, diabetic neuropathy and retinopathy.^(^ ^5^ ^)^ Therefore, the cost per patient increases with time, especially because of the presence of later complications.^(^ ^6^ ^)^

Considering this scenario, the impact of visual loss in individual’s life is devastating, periodic visits to the ophthalmologist are fundamental for diagnosis and early treatment of eye diseases.^(^ ^7^ ^,^ ^8^ ^)^

Public policies such as the statement 957/2008 consider ophthalmologic care as important part of actions for promotion, prevention, treatment, and recovery of quality of life in all levels of health care.^(^ ^9^ ^)^ This type of care can be applied to all health care levels, but it is expected that secondary care considers the intermediate technology density between primary and tertiary care.^(^ ^10^ ^)^ However, the perception of a possible gap in resolubility of ophthalmologic care for patients at a health integrated center in State of Sao Paulo led to the following questions: Is there resolubility in ophthalmologic care provide, specially to diabetic patients? What are the main reasons to request ophthalmologic assessment? What is the waiting time for patients to be attended in the public integrated health center?.

For this reason, emphasizing that one of most important elements for nursing professionals and their role on participation policy and social control of the Unified Health System constitutes the healthcare organization that is responsible for thinking and plan services that are able to respond to the population demand.^(^ ^11^ ^)^ This paper evaluated finding related to ophthalmologic care and compared the results with those reported in previous national and international studies.

## OBJECTIVE

To evaluate resolubility of ophthalmologic care in an Integrated Health Center.

## METHODS

This was a descriptive, exploratory, cross-sectional and quantitative study conducted in an Integrated Health Center in the countryside municipality of the São Paulo State from November 2013 to November 2015.

In July 2013 started to be offer in the health center the care in different medical specialties in addition to physiotherapy, odontology, nursing care, consultations, small procedures, and laboratorial diagnostic tests.

After November 2013, a total of 40 ophthalmologic consultations are done monthly, in addition to payment of specialist physicians to perform four pterygium surgeries, four procedures of retinal photocoagulation with laser and four procedures of YAG laser capsulotomy after cataract surgery.

We included in the study 816 patients who underwent ophthalmologic care. A total of 920 consultations met the following inclusion criteria: to have had an ophthalmologic consultation, and had requested ophthalmologic assessment after medical consultation. We excluded patients who were impossible to reach because of changes in address or phone and death or who had double registration for repetitive consultation reasons. Participants were divided into two groups: Diabetic Group and n Non-Diabetic Group.

After consultation of the service database and, after consultation of patients’ medical record, participants’ sociodemographic status information and characterizations of ophthalmologic care of the center were completed.

A non-probabilistic sample was adopted using quantitative criteria for data collection and processing. Initially, all variable were analyzed descriptively and tables were presented, including absolute and relative frequencies. To compare means of two groups, we used Student *t* test and the Mann-Whitney non-parametric test when supposition of normality was rejected. To assess homogeneity between proportions, we used χ ^2^ test or Fisher’s exact test. Significance level for tests were 5%, the SAS System version 9.4 was used for statistical analyses.

This research project was approved by Ethical Committee in Research involving humans of the Medical School of *Universidade Estadual Paulista “Júlio de Mesquita Filho”* , Botucatu, São Paulo, Brazil, number 1.227.288, CAAE: 48082015.9.0000.5411.

## RESULTS

Most of patients were women (61.9%), white (92.0%), had a partner (75.7%), had completed at least high school (65.1%) ( Table 1 ). Studied population age ranged from 18 years to 94 years with arithmetic mean of 54 years in Diabetic Group and 41 years in Non-Diabetic Group.

**Table 1:** Sociodemographic characteristics of the sample

Variable	Group		Total	p value*

	Diabetic patients	Non-diabetic patients		
Sex				
Female	124 (63.9)	379 (60.9)	503 (61.6)	0.4554
Male	70 (36.1)	243 (39.1)	313 (38.4)	
Formal education				
Elementary	22 (11.3)	140 (22.5)	162 (19.9)	<0.0001
High school	151 (77.8)	380 (16.4)	531 (65.1)	<0.0001
Higher education	21 (10.8)	102 (16.4)	123 (15.1)	0.6943
Skin color				
White	163 (84.0)	546 (87.8)	709 (92.0)	0.2176
Non-white	31 (16.0)	76 (12.2)	62 (8.0)	
Marital status				
With partner	188 (96.9)	430 (69.1)	618 (75.7)	<0.0001
Without a partner	6 (3.1)	192 (30.9)	198 (24.3)	

Results expressed as n (%). * p<0.05; Student *t* test/χ^2^ test.

Of the sample, 362 had associated diseases, and most prevalent was cardiovascular diseases (39.9%), eye diseases (2.4%) and joint diseases (0.6%).

To the majority of patients in both groups, the reason for referrals ( Table 2 ) was general ophthalmologic assessment (77.2%), with significant statistically difference (p<0.0001). However, important facts were the fund evaluation (37.6%) in Diabetic Group and changes in visual acuity (13.7%) for Non-Diabetic, both statistically significant (p<0.0001 and p=0.0132, respectively).

**Table 2:** Reasons for ophthalmological consultation

Variables	Group		Total	p value*

	Diabetic patients	Non-diabetic patients		
Ophthalmological evaluation	107 (55.2)	523 (84.1)	630 (77.2)	<0.0001
Change in visual acuity	13 (6.7)	85 (13.7)	98 (12.0)	0.0132
Fundus evaluation	73 (37.6)	3 (0.5)	76 (9.3)	<0.0001
Other complaints	1 (0.5)	11 (1.8)	12 (1.5)	0.3553

Results expressed as n (%). * p<0.05; Student *t* test/χ^2^ test.

In a comparison of both groups the waiting time for participants to have a ophthalmological evaluation ( Table 3 ) had a statistically significant difference in relation to waiting time lower than 6 months (p=0.0268), being observed that non-diabetic patients wait less time. We also observed that most of diabetic patients wait for a period ≥12 months for a consultation with a specialist.

**Table 3:** Waiting time of participants for ophthalmological evaluation

Waiting time (months)	Group		Total	p value*

	Diabetic patients	Non-diabetic patients		
0-6	26 (13.4)	130 (20.9)	156 (19.1)	0.0268
7-11	79 (40.7)	251 (40.4)	330 (40.4)	0.9941
≥12	89 (45.9)	241 (38.7)	330 (40.4)	0.0923

Results expressed as n (%). * p<0.05; Student *t* test/χ^2^ test.

Of 816 patients attended in our study, 241 (29.5%) were referred to ophthalmological care after consultation in this integrated health center. Of these, 30.7% had diabetes and the highest percentage was attributed to non-diabetics (69.2%) with statistically significant difference (p=0.002) ( Table 4 ).

**Table 4:** Referral for ophthalmological care in network after consultation in a Integrated Health Center

Group	Referral for ophthalmological care			

	Yes	No	Total	p value*
Diabetic patients	74 (30.7)	120 (20.8)	194 (23.7)	
Non-diabetic patients	167 (69.2)	455 (79.1)	622 (76.2)	0.002
Total	241 (29.5)	575 (70.4)	816 (100)	

Results expressed as n (%). * p<0.05; Student *t* test/χ^2^ test.

Among difficulties to perform ophthalmological care suggested by the specialist in other sites of the network (70.6%) we observed that participants were still in the waiting list to do the suggested treatment, however, without statistically significant difference among both groups ( Table 5 ).

**Table 5:** Difficulties to receive health care

Variables	Group		Total	p value*

	Diabetic patients	Non-diabetic patients		
Difficulties for care				
Yes	39 (59.1)	78 (54.9)	104 (50.0)	0.6797
No	27 (40.9)	64 (45.1)	104 (50.0)	
Reasons for not providing care				
To be in a waiting list	24 (77.4)	36 (66.7)	60 (70.6)	0.4237
Refused the suggested care	2 (6.5)	5 (9.3)	7 (8.2)	0.9654
Others^†^	5 (16.1)	13 (24.1)	18 (21.2)	0.5571

Results expressed as n (%). * p<0.05; Student *t* test/χ^2^ test; ^†^ second medical opinion reported the lack of need for eye care; broken equipment and returned to waiting list; patient showed up at the wrong address; mother did not know the medical request; unknown treatment indication.

## DISCUSSION

The high predominance of women in this study, similar to other studies in ophthalmology conducted in Brazil,^(^ ^12^ ^-^ ^14^ ^)^ differs from other countries in which sociocultural and economical differences limited the access to this population for ophthalmologic care.^(^ ^15^ ^)^ As an example we could mention is Africa in which lack of access to and use of eye care services are, perhaps, the main reasons for alarming number of blindness cases among women.^(^ ^16^ ^)^

Formal education level of most participants, who had completed high school, was a different finding from other studies in which most patients who sought eye care had had low level of formal education.^(^ ^13^ ^,^ ^17^ ^)^ This finding lead us to reflect on possible changes in profile of patients in the Brazilian Unified Health System (SUS - *Sistema Único de Saúde* ).

In addition, the highest education level and marital status as “with a partner” that was attributed to Non-Diabetic Group was related to fact that member of this group are younger than Diabetic Group – and not because they were diabetic. In relation to clinical characterization of patients, individuals attended in an ophthalmological care service of Integrated Health Center had associated diseases, and the majority reported to have *diabetes mellitus* and cardiovascular diseases. These findings corroborate with results reported in another study on eye health condition in Brazil that attributed *diabetes mellitus* as a determining factor for vision loss.^(^ ^18^ ^)^

Still, the Diabetic Group included the most of referrals to fundus evaluation what would be solved in primary care if the service had resources, in addition to a qualified general physician to do the exam.

Many referrals did not have more information in each case which meant weak reference used by professional. This corroborated with a previous study that reported that a general physician would hardly be qualified to do a fundus examination under mydriasis, which is most accurate method to detect diabetic retinopathy with consequent low resolubility and inadequate referrals.^(^ ^7^ ^)^

This reality also emerged in Ira where viability of ophthalmologic evaluation was questioned about reference and contrareference, pointing out that in most cases treatable blindness can be avoided by development of preventive or therapeutic strategies. The *diabetes mellitus* is an issue of special concern.^(^ ^19^ ^)^

In Mexico there is also a reinforcement on the need of referrals that include adequate information to continue care in other care levels.^(^ ^20^ ^)^ In addition to be basic premise of guarantee integrality, it is noticeable that integration of care levels are able to avoid duplicity in infrastructure and services, reduce costs and improve resolubility.^(^ ^21^ ^)^

In Brazil, more than half of expenses in ophthalmologic services in SUS cover only costs of procedures related with cataract surgery. Ophthalmologic primary care is almost impossible to solve 90% of main causes of visual impairment with a budget lower than those available only for more complex procedures.^(^ ^7^ ^)^

Based on reality of conditions of care in basic health units of the studied municipality, we observed that such units do not have materials and/or basic equipment required for ophthalmologic screening, *e.g* ., of Snelle table, and much less of an trained team for efficient screening, which justified that reason of higher reference of referrals was the ophthalmologic assessment.

This insufficient and fragile services in Brazilian health care were fragmented and without the infrastructure needed to solve problems contribute to low performance of services, to difficult the access, to the continuing of care, and to the low resolubility and non-optimization of resources available for treatment of patients who need secondary ophthalmologic care.^(^ ^21^ ^)^

For this reason, to share care with other trained professional can be an alternative to integrate and solve care, separating cases that really need assistance from an ophthalmologist. We believe that matrix support also called matrix practice, understood as “specialized technical support that is offered by interdisciplinary health team in order to enlarge the working field and quality their actions” emerges as transforming tool, not only for health and disease process, but to all reality of these teams.^(^ ^22^ ^)^

Therefore, questioning about health public policies appears, and this position the ophthalmologic service in the specialized care, *i.e.* , in secondary and third levels of complexity focused on resolution of prevalent diseases, putting aside the promotion of primary eye health care.^(^ ^7^ ^)^

We can highlight that most of participants who were non-diabetic wait less time for consultation compared with Diabetic Group, which wait for at least 122 months for an ophthalmologic consultation. Currently, half of these patients are still waiting for resolubility in SUS.

This lack of priority of ophthalmologic care certainty compromise the prevention and control of some avoidable cases of blindness and visual impairment.^(^ ^7^ ^)^ Although hierarchy principle used by SUS seeks to guarantee the citizens the access to public health system services from simple to complex care.^(^ ^18^ ^)^ Our study showed that most of patient from both studied group faced difficulties to receive care proposed by the ophthalmologist, and the main complaint was the time on waiting list.

The problem of long time waiting for booking a consultation in health units occurs for a long time; waiting list is a problem that is on population agenda.^(^ ^23^ ^)^ Limitations to access care emphasizes the long waiting lists and lack of satisfaction. Part of population is unable to get care and this exposes users to health risks and may lead them to experience fear and embarrassment.

In Brazil, the waiting list is mentioned as “entrance door” to the SUS and it can impose implication in user’s view,^(^ ^23^ ^)^ but this is observed in access to health service provided for millions of Brazilians who seek a health care service provide with dignity and resolution.

Different from this finding, a study in Iran did not show a waiting list for patients undergoing first clinical appointment, in that study patients were often assisted within 7 days. However, there was no specific plans to improve quality, productivity, efficiency or prevention of blindness, in addition to lack of guidelines for surgical treatment referral, when necessary.^(^ ^19^ ^)^

For this reason, we pointed out the agility in processes as determining factor for quality in service delivery and, for such, ending the waiting list that exist in Brazil is an issue that needs to be done. This conflict is highlighted when demand is restrained without appropriate classify users.^(^ ^24^ ^)^

Other study in ophthalmologic area reveals that problem with difficult to access eye care in SUS is beyond insufficient funding, mentioning, as example, the United States of America, which although has higher finance resources than Brazil, face similar problems because of the way they organize Health Care System.^(^ ^25^ ^)^

In this sense, there is a need of continuous education and new alternatives that enable effective implementation of eye health system that is able to provide continuous care to the population, mainly for those considered as priority cases, i.e., at ophthalmological risk, and by establishing appropriate referrals to care level and on adequate time for resolubility.

Comparison of our study with other existing scientific reports on ophthalmologic care shows that challenges on health management are not solely of Brazilian public health system. This fact reinforces the need of full range of care options that organize and enlarge access to ophthalmologic care by using an integrated care network system including public and private sectors and by focus on continuity of care for patients and on their basic constitutional right: health care.

## CONCLUSION

This study results allowed determining the existence of partial conditions of resolubility to meet real ophthalmologic needs of *Sistema Único de Saúde* users.
